# Multidisciplinary exhibit design in a Science Centre: a participatory action research approach

**DOI:** 10.1080/09650792.2017.1360786

**Published:** 2017-08-20

**Authors:** Hannah Rudman, Claire Bailey-Ross, Jeremy Kendal, Zarja Mursic, Andy Lloyd, Bethan Ross, Rachel L. Kendal

**Affiliations:** a Centre for Coevolution of Biology and Culture, Department of Anthropology, Durham University, Durham, UK; b Department of English Studies, Durham University, Durham, UK; c Centre for Life, Newcastle-upon-Tyne, UK

**Keywords:** Participatory action research (PAR), co-design, informal science education, multi-disciplinarity, impact

## Abstract

In this paper we highlight the issues and opportunities of a participatory action research (PAR) and co-design project, currently being undertaken as engaged research between academics at Durham University and practitioners at the UK’s International Centre for Life in Newcastle-Upon-Tyne (CfL; see creativescienceatlife.com for more information and developments). The focus is on the use of PAR to enable university researchers and Science Centre professionals to co-design Informal Science Learning exhibits that enhance creativity and innovation in young people. We define the principles of PAR and explore reasons for adopting the approach. An account is provided of the iterative co-design and piloting of a novel exhibit within a new exhibition space at the CfL. Reflections collated independently by the practitioners and the academics involved highlighting the development of ideas and insights over the course of the PAR process. We discuss how PAR enabled effective engagement with and creation of enriched knowledge, and innovation, in both the academy and science-learning professionals. The added value of PAR and co-production to our project aligns with current calls for a redefining of how societal impact of academic research is considered.

## Introduction

Participatory action research (PAR) is a framework increasingly used in educational research to achieve good communication, cooperation, collaboration and trust between stakeholders (Lennie and Tacchi 2013). These criteria are essential for improving and encouraging learning, innovation, and for developing responsiveness to different attitudes and values. This article aims to contribute to discussion of PAR as an approach for university researchers and science educators to collaborate and co-produce innovative Informal Science Learning exhibits for visitors, especially young people. Informal Science Learning practitioners develop their activities in order to improve people’s confidence around science, their understanding of the scientific approach, and their appreciation of the results of scientific enquiry. Many practitioners, however, have an exclusive focus on the sciences, with the result that a great many people who prefer the arts, humanities or sports are turned off at a young age by a subject that they cannot personally identify with (DeWitt, Archer, and Osborne 2013). Moreover, there is currently a dramatic downturn in science uptake in UK schools and universities (Macdonald 2014).

Researchers at Durham University and Science Centre practitioners at the UK’s Centre for Life (CfL; Newcastle) are investigating whether developing Science Centre exhibits that use creativity and innovation as alternative forms of enquiry offers a different route into science that can also incorporate other subjects. We formed a multidisciplinary team to co-produce exhibits that enhance creativity, innovation and scientific thinking to examine this notion. Traditionally, Science Centre exhibits are produced and researched in a linear way: developed by Science Centre practitioners, realised and built by designers, and then researched by academics (and reflected upon by the practitioners). To proceed by the traditional production method would have taken a great deal of time, as the academics would have had to wait for the practitioners and designers to first complete the development and design stages before research could even begin. Moreover, such an approach would have lacked the benefits of bringing the ideas and experience of both practitioners and academics together in the inception of the exhibit. We decided on a co-production approach to enable a co-design environment where we could iteratively test our ideas quickly, and rapidly develop practical and theoretical knowledge about whether alternative forms of enquiry offers a different route into science. PAR was the framework for the team’s collaboration, chosen as it is especially appropriate for sectors such as Science Centres where experiential learning and reflective practice are norms. It also has a good fit in a co-design environment where a team undertakes a sequence of iterative events – collaborative cycles of planning, acting and evaluating. The team of practitioners and multidisciplinary academics used PAR as an approach to actively engage together in the quest for understanding about whether alternative forms of enquiry such as creativity and innovation might guide future developments in Science Centre exhibit design.

Following a brief background to informal science learning and the theoretical underpinnings of a PAR approach, we focus on an account of action research carried out by a multidisciplinary team of Science Centre practitioners and academics. The aim was to create novel exhibit(s), situated in the Centre for Life, which simultaneously engaged visitors in the scientific process, while enabling the collection of ethically consented video data for an experimental analysis of creativity and innovation and social learning in children. We report on the exhibit design and piloting process, highlight planning, activity, and independent reflections made by the science centre practitioners and the academic researchers. Of particular interest is the reflective consideration of how engaging with science centre practitioners influenced the academic research process and how engaging with academics influenced the work of informal science learning practitioners. The findings contribute to the practice of research, and in particular how to generate effective multidisciplinary collaborations.

## Informal science learning

Traditional formal education undoubtedly plays a critical role in supporting science, technology, engineering and mathematics (STEM) learning, yet many people are turned off STEM subjects at a young age as they cannot personally identify with them (DeWitt, Archer, and Osborne 2013). Science uptake in UK schools and universities has experienced a dramatic downturn over the last two decades as fewer pupils take STEM as subject choices beyond compulsory education (Lyons 2006; McWilliam, Poronnik, and Taylor 2008), and STEM is still male-biased (Warrior 2002; Halpern et al. 2011). This has resulted in skills shortages in STEM sectors, representing a threat to the UK’s capacity for growth (Straw and MacLeod 2013; Macdonald 2014). This is particularly evident in North East England, where the case study Science Centre is located, and there is a relatively low uptake (particularly among females) of science, technology, engineering and mathematics subjects (NELEP 2014). The North East also suffers the lowest progression rates to higher education in England, which has led the UK Commission for Employment and Skills to project that by 2020 there could be significant North East regional shortages of high level STEM skills (UK Commission for Employment and Skills 2014). A potential key factor in this decline is a perception that science is not a creative endeavour. Surveys of student and community attitudes consistently identify rigid, dogmatic thinking as characteristics seen as essential for success in science (Barak and Shachar 2008; Barton, Tan, and Rivet 2008; Schmidt 2011). It is therefore increasingly important to emphasise the contributions of informal science learning environments, including but not limited to Science Centres and Science Museums that strive to redress this by encouraging creativity and experimentation with the aim of ensuring access for all to inspirational science. This mirrors the growing awareness that an individual’s direct, personal experiences, needs, expectations and cultural background significantly affect attitudes toward, and understandings of, science (Falk, Storksdieck, and Dierking 2007; Zhang, Schmader, and Forbes 2009; Dierking and Falk 2010).

Academic researchers in child development are steadily gaining new insights into the intricacies of children’s reasoning and scientific thinking (Gopnik 2012). Piaget originally argued in favour of discovery learning and exploration in children (Inhelder and Piaget 1958). The constructivist idea is that discovery learning, in opposition to direct instruction, is the best way to familiarise and gain understanding of scientific principles, especially in young children (Hein 1999; Bonawitz 2011). We understand discovery learning as a minimally guided pedagogical approach (Kirschner, Sweller, and Clark 2006), where children develop or construct knowledge on their own (Klahr and Nigam 2004). Scientific creativity is a distinctive type of creativity that is usually missed in formal educational environments such as schools (Newton and Newton 2010). Science Centres then, visited informally in leisure time or in school trips, are a perfect place to facilitate scientific curiosity, exploration, and scientific creativity. There has been increased recognition of the important role that visits to informal learning institutions play in supporting science learning. To date, however, most informal science education impact studies have been conducted with adult visitors (Sandifer 2003; Falk, Storksdieck, and Dierking 2007; Falk et al. 2016). Our *Design for Creativity and Innovation in Informal Science Learning* project, discussed in this paper, specifically focuses on young people.

Traditionally, academic research and Science Centre practice unfold independently with different aims, objectives, and methods. The current project is our first attempt at co-produced research that simultaneously advances academic research and societal engagement with science. The aims of the collaboration align with recent work of *The Centre for Advancement of Informal Science Education* (CAISE) which, via the ‘Research + Practice Collaboratory’, has successfully applied co-design and design-based implementation research methods and made available a series of openly available online resources (researchandpractice.org) for educators and practitioners when research is being undertaken in formal education settings. Simultaneously, there have been recent calls for the broad field of informal science education research (ISE) to embrace collaborations and boundary encounters, so as to learn from other subjects (Martin 2004; Rahm 2014).

The multidisciplinary co-production of research outcomes and societal benefits is consistent with the UK Government’s Research Excellence Framework (REF) exercise. This requires the submission of impact case studies that have ‘any effect on, change or benefit to the economy, society, culture, public policy or services, health, the environment or quality of life, beyond academia’ (HEFCE, SFC, HEFCW, and DELNI 2012). Following REF 2014, the Higher Education Funding Council of England stated that impact ‘often stem(s) from multidisciplinary work and reflect(s) the way that universities have engaged with a range of public, private and charitable organisations and local communities’ (King’s College London and Digital Science for HEFCE 2015).

Similarly in the US, National Science Foundation (NSF) funded projects are awarded extra merit for meeting ‘Broader Impacts’ criterion and it is recognised (Sacco, Falk, and Bell 2014) that partnering with Science Centres is a valid means of achieving impact. A recent N8 / ESRC Research Programme report identified the paradox of co-produced and participatory research being highly effective in generating impact whilst simultaneously not being recognised, or facilitated, in current requirements of impact reporting or evaluation (Pain and Raynor 2016). In contrast to co-production of research, the traditional ‘donor-recipient’ model of impact, requires a single knowledge producer (University/academic) to impact on economy or society in a linear fashion. With our aim of co-produced research and impact, with, rather than for, an organisation and its community, we adopted the PAR process.

## Principles of PAR

The principles of PAR originated over 70 years ago with Lewin and the Tavistock Institute (Lewin 1946). It is practice-led, rather than practice-based, and contrasts with traditional scientific research where participants are objects of the study. The PAR approach typically helps to create actionable knowledge. PAR demands that research takes place concurrently whilst action is ongoing, with research and actions undertaken by the participants. The responsibility for theorising and solving issues does not rest solely with the academic, it is collaborative – participants of the system being studied are actively engaged in a cyclical process of planning, acting and reflecting. It is this idea of meta-learning through the inclusion of academic and practitioner reflection, that elevates action research above every day problem solving (Schon 1983; Argyris 2003). Our team of academics and practitioners require different outcomes from the development of the Science Centre exhibit, and PAR enables this: the academics want to create new academic knowledge concerning creativity and science engagement (which they can research through an exhibit in a Science Centre); the practitioners want to know how to engage more people with science at the centre through exhibits that offer different approaches (but they do not yet know what those approaches are – research is required). PAR practitioners attempt to integrate three aspects: participation (life in society and democracy), action (engagement with experience and history), and research (soundness in thought and the growth of knowledge; Chevalier and Buckles 2013, 6 and 8) with practical actions seamlessly uniting with research (Chambers 2008, 315) and typically being performed ‘with’ people and not ‘on’ or ‘for’ people (Chevalier and Buckles 2008, 5). The academics and practitioners are participants undertaking a sequence of events – collaborative cycles of planning, acting and evaluating – actively engaging together in the quest for information and ideas, that might guide future actions. The approach includes collective fact-finding, analysis, and decision-making involving egalitarian participation by a team to transform some aspects of its situation or structures, through action, research and experience (Reason and Bradbury 2008, 1; Coghlan and Brannick 2010). PAR can be particularly effective for multidisciplinary research. PAR approaches focus on enabling full participation of all those involved in the research process (Shura, Siders, and Dannefer 2011) and forging partnerships so participants can explore possibilities for transformation together (Frisby et al. 2005). The research process is considered to be as significant as the outcome (Pain and Francis 2003). While this might at first appear to be at odds with the usual systematic research process, it has been suggested that it does not fundamentally alter the research method: rather, it places it within a process where it is developed and discussed by a group who have a range of perspectives, knowledge and expertise (Lane et al. 2011).

We decided that a PAR approach was appropriate for the team’s collaborative, iterative, co-production of a new Science Centre exhibit which would enable both academics and practitioners to test and understand whether creativity and innovation work as alternative forms of enquiry into science. Traditionally, Science Centre exhibits involve a linear production process with decisions about design being first planned and developed by Science Centre practitioners, then designed and built by exhibit designers, and finally researched by academics. With the PAR approach, we were able to transform this process. We pooled our practical experiences and theoretical expertise at the development phase through participative workshops and meetings where we went through cycles of thinking, planning and acting to iterate the development of the Science Centre exhibit to meet all of our aims. We applied practical and theoretical requirements from multiple relevant disciplines, giving them equal importance at the planning and development and design and build stages. We jointly experienced the effectiveness of the pilot version of the exhibit, and we further discussed and agreed changes to the next iteration so that it met both academic and practitioner outcomes – simultaneously engaging visitors in the scientific process while generating experimental data for scientific research. The benefit of the PAR approach here was that we could test and consider whether the plans and concepts for the new Science Centre exhibit and the ideas for its design worked for: the academics (would it be able to collect the sort of data required for their scientific enquiry?); and for the practitioners (would it be fun and attractive to the Science Centre visitors and engage them in more science?). The research is *in* action, rather than *about* action and the outcomes are twofold: an action and a research outcome (Pedler 2005).

We came together as a team, using ESRC Impact Acceleration Account funding from Durham University, and with the help of the CfL who were working with a number of academics on different projects, which combined, offered the ideal range of approaches, breadth of knowledge and expertise. We found PAR an effective approach for a multidisciplinary team to undertake research and development, because we had to commit to including all disciplines equally. Both the enquiry and decision-making are therefore open and jointly negotiated (see Pain, Kesby, and Askins 2011). We detail our PAR approach and principles, and the cycles of planning, action and reflection that we undertook below, in the Science Centre case study.

## Science centre case study

**Figure 1.**
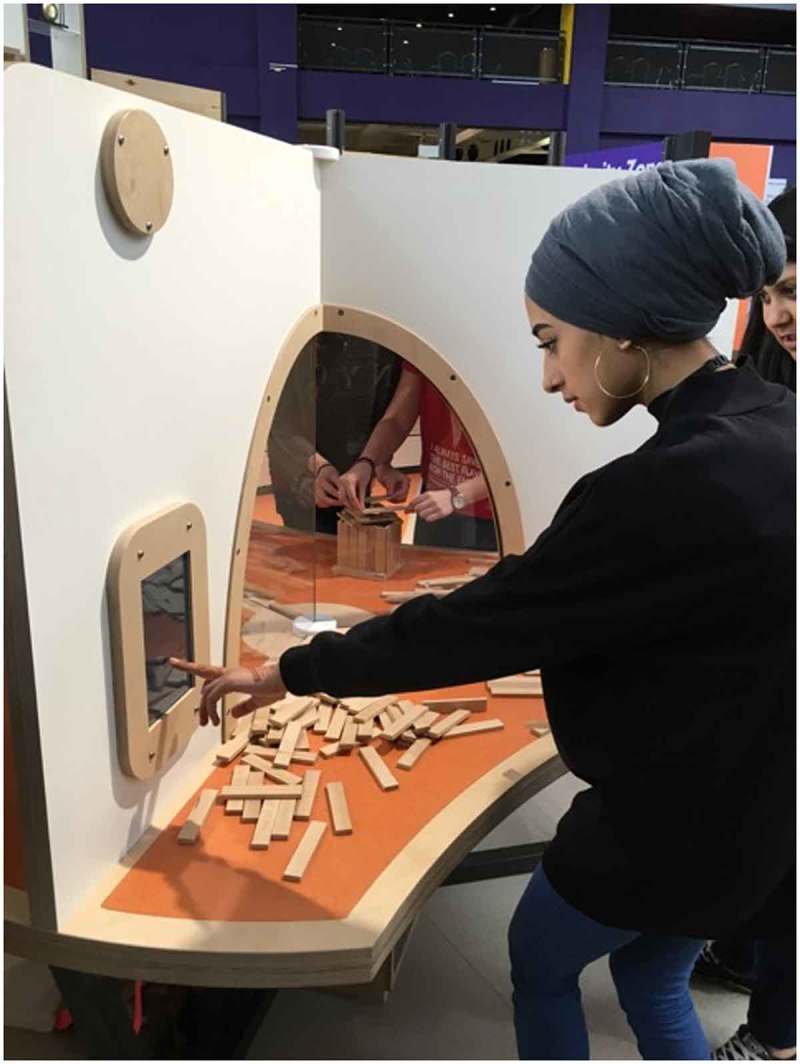
Visitors engaging with the task at the interactive research pod.

The core team consisted of five academics from Durham University (from Anthropology, Information Systems and Digital Humanities), and two science-centre practitioners from the CfL. This paper draws on a single case study – the co-design and development of a new exhibit for the Science Centre; a novel, live experiment forming part of the new Wellcome Trust funded ‘Brain Zone’ exhibition space at the Centre for Life (opened in spring 2016). The Brain Zone focuses on how scientists explore the brain’s inner workings and capabilities. Our ‘interactive research pod’ is an interactive exhibit at which visitors build structures using wooden cuboid shaped blocks (see Figure 1). The pod was designed so that the researchers can compare behaviour under different experimental conditions: participants (visitors) can (i) build alone, (ii) build while observing others’ buildings, or (iii) collaborate on builds. The project aim was to develop an interactive exhibit (or exhibits) that would engage visitors in the scientific process of research, encourage creativity, while also allowing researchers to collect ethically consented video data suitable to compare innovation and creativity across the three experimental conditions. Digital tools embedded in the exhibit allow researchers to gain ethical permission to record build activity and visitor information (e.g. age, sex, etc.) for analysis.

An integral part of the PAR process is the establishment of ‘a community of researchers’ that share ownership of the research process (Cahill 2007). For us, a mix of practitioners and academics, this was the most attractive aspect of PAR over more traditional empirical research approaches (where the academics own the research process, and the practitioners and their work is the subject of it). Our core team included the Science Centre’s Head of Special Projects and Exhibition Researcher; three anthropologists, one digital humanist and one computer scientist. Designers and other specialists joined the team on an ad hoc basis. Our PAR approach was co-designed at the first meeting. We agreed explicitly that every team member had equal voice and democratic influence and access, ensuring that meetings constituted an environment where knowledge was openly shared and transferred. We worked together for two years meeting monthly. The principles and practices for originating, designing, conducting, analysing and acting on our PAR project were as follows:

• Each meeting would have academic and practitioner inputs, such as presentation of data or diagrams, or research, or field trips to see activity by visitors on the Science Centre floor.
• Each meeting involved periods of planning, action and reflection. 
• Each meeting had a group discussion, which would equally address academic and practitioner issues. Every discussion contained reflection which helped narrow our focus to result in agreed outputs and actions to be completed by the team members between monthly meetings. We created a culture of systematic reflection within the project team.
• One team member (neither from the CfL nor the main academic thrust of the project) explicitly took on a ‘communicator’ role, facilitating meetings to ensure equal voice and democratic influence from all of the team.
• Principles were agreed for communication: online project management mechanisms established for sharing timelines and documents; and group email for discussing plans, actions and reflections between meetings.
• Meeting notes summarised reflections and plans, and highlighted actions.

All of this process created outcomes and activity that we further reflected upon each month, developing our PAR approach. PAR has to remain flexible in use. For us, this meant that the approach was suitable even when plans and actions and even research questions changed, as everyone in the team iteratively reflected on the plans and actions. At every stage, the team democratically applied academic theory and professional practice to the main objective of the project: the iterative co-design of novel Science Centre exhibits. Our exhibit is being developed specifically to answer research questions on whether we can encourage creativity and innovation in scientific thinking, and how we can capture and measure creativity in visitor behaviour.

In the following section, we describe in detail how the team worked under our PAR approach. The iterative cycle of planning, action and reflection during the Design and Pilot phases of the project are discussed. Reflections collated independently by the practitioners and the academics highlight how their concerns and insights evolved over the course of the project. The current Live Phase is summarised to highlight the data academics and practitioners are collating to answer the research questions.

## Design phase

During the design phase (January 2015 – September 2015), workshops and meetings were held at which academic theory and professional practice were applied to iteratively design the interactive research pod. Research questions, outputs and objectives were decided democratically between the project team. A key focus was to ensure that the interactive research pod could automatically collect data for the scientific study while being engaging to visitors.

**Figure 2.**
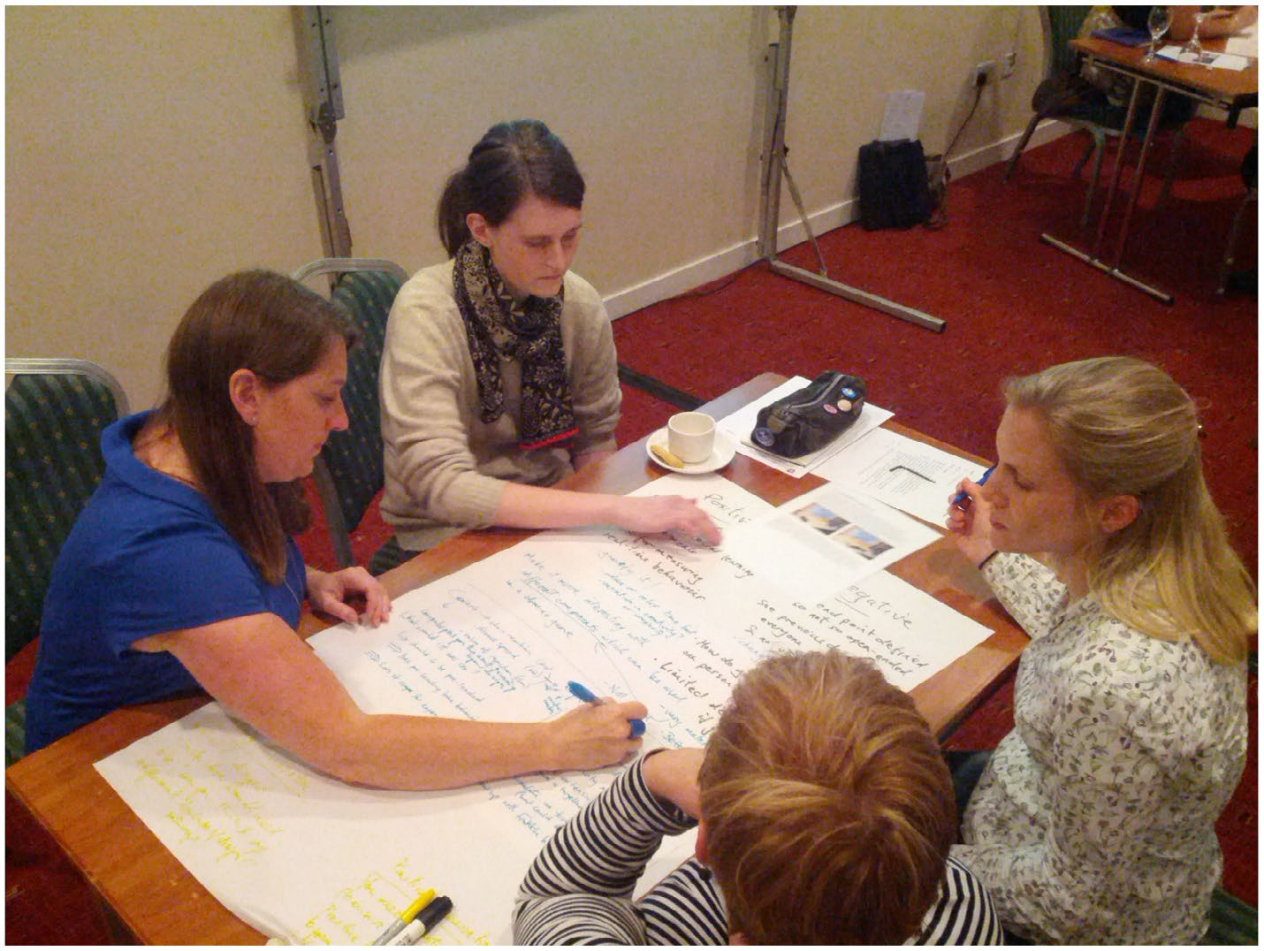
A mixed discipline team brainstorming an exhibit type.

As part of the initial design phase of the project, the core team collaborated with a wider group of external advisors. Exhibition designers from other Science Centres, science interpreters from the CfL gallery floor, and a range of academic researchers (from anthropology, digital humanities, education and psychology) were invited to a co-design workshop with a design-thinking facilitator. Through the workshop we aimed to gain feedback on a shortlist of five exhibit designs, already developed by the project team. The workshop was held early on in the design phase, at a point where the exhibit designs iterated by the project team could be clearly described, but could also still be influenced and improved. Following an introductory session, design thinking exercises were undertaken (Kelley 2001; Brown 2009; Cross 2011). Participants split into small mixed-expertise groups to evaluate each exhibit design (a written description with diagrams presented at separate workstations in the room). The groups discussed the imagined visitor experience of each design (in turn); recording feedback on A1 paper, including positives, negatives, suggested changes or hybrid designs with other proposed exhibits (Figure 2). The overall feedback recorded for an exhibit was then presented and participants voted for their top two exhibits using ‘dotocracy’, by placing dot-shaped stickers on the exhibit feedback sheets they preferred.

Of the five proposed exhibit designs, two received particularly positive reviews, one was discounted, while it was recommended that some aspects of the final two exhibits were blended into one. Workshop participants were then invited to choose which of these three exhibits they were most interested in developing further. These three self-chosen groups worked on defining visitor journey/experience maps for each.

Prior to, and following, the workshop, the core team participated in regular meetings by inputting research-informed knowledge and craft practice know-how through collaborative discussion. Deliberations in each meeting informed the design of the exhibits, the ethical consent gathering approach, the data gathering methods, and honed the research questions. After each meeting, outputs, practical actions and outcomes were recorded (see Table 1). The three top exhibits from the workshop were (i) a digital game to do with creating and keeping creatures alive in their ecology, (ii) a physical building block-type construction across a ravine challenge, and (iii) a task involving digital instructions and a physical creativity challenge (using pipettes and coloured liquid). Our final outcome, being an interactive research pod that could house various challenges, was different to any of these exhibits but drew inspiration from them. The exhibit we chose to house in the research pod was a building task (like exhibit two) but more open-ended to enable creativity and simplify data collection. This building task is physical but we are also piloting a digital version (like proposed exhibit one) in order to compare creativity across the physical and virtual domains. Finally, the interactive research pod itself, was designed to merge digital and physical aspects in one exhibit (like proposed exhibit three), with a digital consent system for visitors to interact with (emphasising their role in active research) prior to taking part in the challenge.

**Table 1.** Researcher and practitioner inputs, and the resulting outputs and outcomes during the design phase.

Meeting date	Academic input	Practitioner input	Outputs	Outcomes
January 2015	• Ideas around research questions that the exhibits might address, and ideas for how this research might create impact • Proposed information systems for gathering data about and managing the PAR process	• Clarifying which research questions would be also interesting to the science centre • Highlighting dissemination channels and publications used by the informal science learning community	A list of professional and academic journals to be targeted	Agreed timelines, and project management document sharing and data collection tools. Agreed aims and objectives for the project. Agreed research questions
March 2015	• PAR process to include input from wider set of stakeholders • Literature reviews from different perspectives proposed • Concern expressed about how to control conditions of the exhibit (ensuring they are equivalent except the variable in question)	• Suggestion that the group designs a ‘pod’ for the exhibit which would allow it to be experienced in different conditions in a very controlled way	A draft agenda for the workshop with the group of wider stakeholders	Agreement on the target conferences, professional and academic journals. Agreement on the desire to develop a pod to enable and control the required conditions
April 2015	• Exhibits were proposed that would create the type of data required for analysis of the research questions • Digital interpretations of the exhibits, and digital information systems for gathering data were proposed	• Types of design conditions that can be varied were highlighted • Examples of similar exhibits to those proposed, and digital interpretations of them, were provided	Long list of 5 exhibits agreed and written up as descriptions. Definitions written up of the terms ‘creativity’ and ‘innovation’	Recognition that gaining ethical consent from participants would be complex, and needed just as much designing and planning as the exhibits and the exhibit pod
May 2015 – wider stakeholder workshop	Anthropology, Psychology, Education Research, Information Systems and Digital Humanities perspectives applied to the exhibit shortlisting	Design thinking approaches and design practices applied to the exhibit shortlisting	Shortlist of 3 exhibits agreed, and worked-up as user journeys and user experiences	Confidence that the 3 exhibits would provide data for analysis around the research questions as well as forming engaging and fun activities in the new gallery at the science centre
June 2015	Solo, social and collaborative conditions for exhibits were clearly defined	Sketches of how the exhibit pod might look, and walk-throughs and sketches of how the exhibits might work in the pod were produced	Detailed briefs for each of the three exhibits developed	Academic research methodology for the exhibits split out from the design briefs
August 2015	• Ideas for how digital information systems could collect data were proposed and brainstormed • Recommendations were proposed about how to collect the data ethically and we discussed what signage would need to be on exhibits, and how consent from participants could be gathered	Proposed that the exhibits should be developed in the order of 1. Physical, 2. Digital, 3. Physical/Digital blend. Piloting of physical and digital exhibits recommended	Detailed brief for the exhibit pod to facilitate both solo and social conditions developed and included in *Brain Zone* gallery design brief	Agreement that the collaborative condition requires a researcher in person to collect ethical consent, as participants are in a larger more chaotic group engaging with the exhibit. Also that the individual and social conditions may function with a remote collection of ethical consent
September 2015	• Additional touchscreen tablets were proposed to both gather remote ethical consent and give task instructions • Wording for exhibit signage and for gathering ethical consent were proposed	• IP surveillance cameras and a network video recorder system proposed as the solution for video data collection. Scale of hard disc drive estimated by working out how much footage would be collected over time	Secure data transfer protocols between the science centre and university agreed. Ethical consent wording agreed and data archiving protocols agreed	• Onscreen ethical consent system approved by University Ethics Committee. Open source survey software will be used as the mechanism for the ethical consent form • The team is ready to pilot the data collection of the physical activity in solo, social and collaborative conditions

### Reflection on the design phase

Four key areas of reflection were identified during independent consideration by practitioner and academic project team members. The uneven balance of reflected topics by practitioners and academics contributes to an appreciation of variation in concerns, or highlights, over the course of the process.

#### Reflection 1. Evolution of interactive research pod design

*Academic reflection*: Discussion with CfL practitioners over design needs, and synthesis with the Anthropology/Psychology research background, led to the idea of each of the three proposed exhibits containing two conditions: individual and social learning. Observations of visitor interactions by practitioners in the CfL’s hands-on Curiosity Zone spurred the idea to include a third condition: collaboration. Linked with the emergence of this three condition experimental design was the practical outcome of designing a single pod structure, for use with all three exhibits, that could be configured, with minimal hassle, for all conditions (individual, social and collaborative learning). The actual design of the pod to facilitate this, with the use of opaque, transparent and removable partitions, respectively, resulted from a joining of academic and practitioner know-how. A standard experimental design for innovation and social learning studies in animals involves the use of partitions to control the opportunity for interaction and observation (Kendal, Coolen, and Laland 2004; Day et al. 2001). This experimental design feature was adopted and tweaked in light of practitioner concerns over visitor needs such as ergonomic considerations (e.g. table height, cut outs in tables to encourage a certain number of people to participate etc.), and practicalities (e.g. reusability of pod and robustness).

*Practitioners’ reflection*: There is often a misalignment between exhibition delivery timescales and the academic research process. Therefore, it was essential to ensure the academics were aware of temporal constraints. In particular, how the research project needed to be integrated into CfL’s exhibition delivery project; the exhibition timescale needed to drive development of the physical interactive research pod and it was necessary to defend this project’s activity when the scope of the overall exhibition was scaled back to hit budget. Thus, it was integral to ensure research project ambitions fitted within practical constraints of the exhibition delivery (e.g. time, budget and available space).

#### Reflection 2. Value of the workshop

*Academic reflection*: The workshop was highly beneficial in ensuring the ideas and plans fit broader fields beyond those covered by Anthropology and Psychology. For example, Education researchers highlighted a different perspective regarding how creativity is defined and the problems in the school curriculum regarding student perspectives of a lack of creativity in science (Newton and Newton 2010). They also confirmed the need for the social learning condition, due to Vygotskian theories of development in children’s learning (Vygotsky 1978), and the collaborative condition, due to the benefits of group or peer learning.

The workshop facilitator, experienced in design thinking, encouraged us to walk through the visitor experience, using visitor personas, while elaborating on the designs of the three highest rated exhibits. This was helpful as it encouraged aspects of visitor attraction, attention, interest, engagement and user experience to be considered equally alongside the research interests of the exhibit design. Likewise, inclusion of practitioners experienced in hands-on exhibit design (e.g. the Curiosity Gallery at the CfL) and psychologists interested in the influence of object ownership on creativity (Defeyter 2014) highlighted necessary practicalities requiring consideration. Finally, a digital humanities specialist (CB-R), who joined the core team, highlighted research regarding digital resources in informal learning contexts such as museums and galleries, for example (Tallon and Walker 2008; Parry 2010; Kidd 2014). They highlighted that digital exhibits to measure creativity (rather than aesthetics) were novel to digital humanities in addition to psychology and anthropology.

#### Reflection 3. Experimental research agenda

*Academic reflection*: It was highlighted that creativity and social learning could be measured using a captivating task familiar to participants, contrary to standard social learning experimental protocols, such as novel puzzle boxes (Carr, Kendal, and Flynn 2015). Practitioner input encouraged a simpler exhibit design than we initially envisaged, that could facilitate visitor engagement and ensure a large sample size for scientific analysis. The practitioners’ preference for a simple design also facilitated interpretation of experiment results. To ensure that the data collection process was also designed to ensure accurate and rigorous scientific research it was important to uphold the value of adequate experimental control within an exhibit between conditions (e.g. the pod housing the exhibit remains the same except for the presence or type of partitions). It is also noteworthy that the scientific experimental design offered a hands-on learning opportunity to bring knowledge not normally covered by arts and humanities, with exciting possibilities for new discoveries and critical confluences of ideas and practices. In particular, the focus is on controlling experiments (conducting only one variable change at a time in order to isolate the results) in an informal learning environment and how this can be achieved.

*Practitioners’ reflection*: The continual discussions raised interesting and useful research questions. It was an important process to minimise the risk of trying to design activities for the other party’s interests. There was a need to compromise between creating an engaging exhibit and detailed scientific interpretation. The academics wanted to incorporate more detailed scientific content than would have been required for a traditional exhibit. There was a need to refocus discussions around interpretation and textual content to highlight to the academics that the amount of factual content that an exhibit (or label) can carry is very limited. The value of the exhibit activity to visitors is that it is an example of learning research rather than anything intrinsic to the building activity itself. It was necessary to emphasise that an open-ended building activity was sufficient for this task.

#### Reflection 4. Ethical consent

*Academic reflection*: Ethical approval was gained from the ethics committee of Durham’s Anthropology Department for remote collection of ethical consent following the British Psychology Society’s (BPS) guidelines. The BPS guidelines were translated into visitor friendly tablet consent systems using the PAR approach of iterations of group meetings and physical piloting on the gallery floor. Digital humanities and practitioner perspectives assisted with ensuring the questions were user friendly and encouraged the filtering out of information that was not strictly necessary for ethical or experimental purposes. The traditional way that anthropologists/psychologists collect video data (with consent and a researcher present) was adapted and the practitioners assisted with this due to their experience of video use in galleries (digital humanities) and visitor perspectives on surveillance (practitioners). For example, covert surveillance is not ethically approved of but the practitioners clarified that visitors would not be put off by clearly visible camera installation.

*Practitioners’ reflection*: We learned about the complexities of ethics approval for publication (compared to typical internal consent requirements for non-published practitioner research and evaluation). The project required compromises on both sides. The ethics system was longer and more complex than we would have hoped; however, the level of control we could ensure was less than the researchers would have hoped for. It was necessary to research suitable products for filming and for consent systems. The consent system stretched the abilities of our in-house team, requiring more programming than expected but proved to be an attractive challenge to the IT manager.

## Pilot phase

Live pilot studies were undertaken on the gallery floor in the Centre for Life during busy school holiday periods (October 2015 and February 2016). We conducted ethnographic research by (i) observing visitor interactions with the digital/remote consent system, (ii) gaining consent in person, and (iii) asking for visitor feedback on the exhibit (especially when piloting the digital version of the building blocks task). Further outputs and outcomes emerged as we continued working iteratively through PAR (see Table 2).

**Table 2.** Researcher and practitioner inputs, and the resulting outputs and outcomes during the piloting phase.

Meeting date	Academic input	Practitioner input	Outputs	Outcomes
October 2015 – piloting the physical exhibit	• Ethnographic evaluation of participants’ interactions with the exhibit in use on the gallery floor revealed shortcomings in the digital ethical consent form, and in the design of the exhibit pod, and in the instruction signage and labelling around the exhibit	• The exhibit in action showed how it would impact visitor flow on the gallery floor, useful to the planning of the final layout of the new *Brain Zone* • Suggestion that unwanted parental interventions happened less when there were seats available around the exhibit	Watching the participants interact with the exhibit and with the exhibit pod and its digital research tools in a live pilot environment provided evidence for feedback. Proof that the system works as an engaging exhibit and as an effective research tool	• Improvements to the pod design, the digital data collecting information systems, the digital ethical consent system and the signage around the exhibit on the science centre gallery floor • The 231 digital data-sets with full ethical consent granted from the pilot study are held securely by the University, and can be analysed by many different disciplines there to answer multiple research questions
November 2015	• Simplified ethical consent wording created to make participant interaction with it easier (especially children)	• Research into digital games and apps that mimic the activity in the physical experiment reveals that we will need to commission a bespoke app development	• The digital exhibit brief is developed • A list is written-up of iterations needed to improve the digital ethical consent form, signage and design of the exhibit pod	The final plans for the new permanent *Brain Zone* reflect what has been learnt from the piloting of the exhibit
December 2015	• Video footage from the pilot reviewed to ensure it can be coded in order to answer research questions regarding creativity and social learning • Analysis of the pilot study shows the impact of PAR as an appropriate approach to co-design and co-research	• Practical considerations are suggested regarding how the collaborative condition of the exhibit can be managed with partitions surrounding the exhibit. Partitions can be removed for solo and social conditions to increase visitor flow	All improvement iteration ideas in final version of design brief now with gallery and software designers	First publications for professional practice journals are written, following the learnings from the pilot and conclusions these provided
January 2016	• Reflection that the end result of what visitors build is important to collect becomes an additional brief for the digital version of the block-building exhibit, and for the process of data collection of the physical version • The collaborative condition of the digital experiment will need to mirror the physical experiment and take place on a horizontal interface	• A multi-touch 80″ screen with closed steel chassis and reinforced glass is recommended for the collaborative condition of the digital experiment. The February pilot will need to test how it copes with glare from overhead lighting	• Agreed wording for signage around the exhibit, explaining that live science experiments are taking place • Agreed wording for next iteration of ethical consent form • Revised feedback for software designers produced	• Publications shared with academic and practitioner colleagues begins to build interest in the exhibit and experiments, and the process of developing it • Confidence is built up in the team that we can invite VIPs and funders to the *Brain Zone* (and hence our exhibit) launch
February 2016 – piloting the digital exhibit and second pilot of physical exhibit	• In depth review of the first iteration of the digital version of the experiment. We recognised that the physical and digital experience can’t be exactly the same, but the outcome of the activities can be	• Design constraints on the digital version recommended, from experience with other digital exhibits	• Another iteration of the ethical consent form • Another iteration of the brief for the digital exhibit	The second pilot highlighted that improvements to ethical consent and data collection were viable, and gave an idea of numbers of participants we can expect at peak times
March 2016	• Watching participants in the second pilot highlighted that people are still trying to take pictures of their constructions with the cameras • A review of the CSV download of the ethical consent form responses (and times taken) revealed which questions were too hard or misunderstood	• Recommendation that the build screen on the ethical consent form could prompt the participant to take a photo of it with the tablet’s front facing camera • Further testing of the progress of the digital version of the exhibit reveals further issues	• Creativescienceatlife.com project website launched • Tweak list for the ethical consent form • Tweak list for the digital exhibit	Other academics working on creativity in science and on young people in informal science learning environments can see and understand what we are doing, and closer relationships with others’ related projects are developed
April 2016 – live launch of the exhibit	• In person explanations to funders and VIPs at the launch generates interest	• A successfully working exhibit with signage and congruence with the rest of the Brain Zone gallery is launched, and VIPs and funders are guided around it and shown its potential through students being *in situ* during the tour	• Live, launched exhibit in the new Brain Zone gallery, visited by VIPs, the original workshop attendees, funders and the public • Journal paper prepared	Funders express interest in supporting further research using the exhibit as an engaging, live science experiment

### Reflection on the pilot phase

Three key areas of reflection were identified.

#### Reflection 1. Future costs of the exhibit

*Practitioners’ reflection*: The pilot phase provided us with evidence to defend the exhibit from potential gallery budget cuts. The pilot also enabled us to assess what the operational load of this sort of installation might be. From the pilot testing we were able to conclude that the final version should require little or no on-going attention apart from when the experimental condition is changed (insertion/removal of partitions).

#### Reflection 2. Digital ethical approval system

*Academic reflection*: We discovered that certain aspects of the consent form were too complex (as indicated by dwell times on these questions, or queries when gaining consent in person) and the need for simplification. We were also pleased to see participants’ desire to provide relevant information about themselves or their child (e.g. autism spectrum disorders, player of Minecraft/Lego) prompting us to include an optional free-text question in the final consent system.

*Practitioners’ reflection*: More visitors persisted and completed the ethical consent form than we had expected, challenging some of our assumptions about visitor behaviour. This alleviated our worries about the length of the consent system questionnaire.

#### Reflection 3. Visitor perceptions and feedback

*Academic reflection*: It was determined that simple building blocks were interesting enough to compel individuals to engage with the exhibit and build something of sufficient complexity, originality and diversity to measure creativity/innovation. The participants’ desire to inform researchers of what they had built indicated that we could collect this information using the consent tablet to enrich our assessment of creativity. Finally, on a practical front, participant behaviour indicated the need to mark out a build zone within each workstation at the pod to ensure the builds were fully captured by the video cameras.

*Practitioners’ reflection*: We were pleased to see that the very act of labelling the exhibit as an ‘experiment’ changed adult visitors’ perception of the activity; this was not considered just a basic building blocks activity (only suitable for nursery children), but a learning research experiment that everyone could take part in. It seemed that the ‘research’ label made the activity acceptable.

## Live phase

The interactive research pod has been operating live at the CfL, in The Brain Zone exhibition, for a year, automatically gathering ethical consent and video data of visitors’ creative interactions with a science task. During this phase, the data collected (5500 + visitors) will inform academic scientific studies on human behaviour in the field of anthropology. The sample is diverse, and the non-laboratory condition of the gallery floor gives the data high external validity, with the high sample size countering the lack of experimental control, or internal validity. We have also collected 120 interviews as well as visitor survey responses, providing useful data for both the academics and practitioners in gauging the success of the exhibit from the general public’s perspective. Preliminary results from these data indicate the general public’s enthusiasm for research involvement, and the potential for the interactive research pod to engage many in science. (Reflections from this phase are still to be collected).

## Discussion and conclusions

The main purpose of this paper is to reflect upon the extent to which PAR, and co-creation of exhibits in a multidisciplinary team, resonates with Science Centre exhibition development, and to explore the potential benefits of the PAR process for use by academics and Science Centre professionals (or other non-academic organisations) in the future. Three months after the Brain Zone exhibition opened, the interactive research pod had collected data from over 1000 participants with ethical consent. Our reflections and early analysis suggest that the interactive research pod exhibit successfully engages visitors in the scientific process while simultaneously providing an effective research tool for academics. While we feel that PAR has been an effective approach for our multidisciplinary, collaborative project between academics and Science Centre practitioners, it is also important to consider the challenges of implementing PAR projects in this context. In the following, we highlight some of the themes that have emerged, and propose means by which the informal science learning and academic sectors might tackle them to improve the prospects for PAR projects in the future.

### Timescales and scope creep

With any participatory project there is always a concern that the extra time and effort required to work in this way may not produce a valuable output that is deemed to be sufficiently different from other exhibits. In order to successfully develop PAR projects in informal science learning environments it is important that realistic timescales are adopted for all project partners. Developing exhibits from scratch can take a significant amount of time, and not allowing for this in predicted timescales may have a detrimental knock-on effect on innovations in exhibit design at later stages in the project. It is also important to ensure that the project scope is achievable and not to be afraid to pare back the original idea if required. Unrealistic scope and timescales mean missing deadlines, which can affect the benefits of the research leading to a lack of opportunity to feed the project findings back into the research and development process. In our case, we originally planned for three exhibits but reduced the scope to one simple block-building activity and focused instead on an interactive research pod that could flexibly house a variety of activities in the future.

### Effective communication

From the outset, the project team aimed to be as open and transparent as possible and stressed inclusion of all, including the Science Centre visitors, in the design and pilot processes. We suggest that buy-in from the CfL CEO and Board of Trustees from the beginning of the project empowered the project team, giving them leeway, freedom and authority to make decisions. Having to secure institutional permission at every stage in the process would have been a barrier to innovation and development. Competent decision-making goes hand in hand with good channels of communication. Clear, regular and transparent communication is required, not only externally but internally. Allocation of a ‘communicator’ role (who compiled minutes and action points) and use of a server that all the core team had access to ensured all parties received automatic notification of any changes, and were able to react and continue to provide equal input into the project. This was particularly important because what might seem unimportant to one party may have a significant impact on the ability of the other project members to complete tasks.

### Democracy

The relatively small size of the project team may have facilitated democratic participation. Moreover, neither the practitioner nor academic viewpoints took precedence. This is despite the fact that the academics outnumbered the practitioners and may be due to the decision to have a designated ‘communicator’ who was able to independently glean, and record, the key outcomes and actions of meetings. However, the personalities of those involved and prior working relationship of practitioners and academics undoubtedly contributed to success in this area. Successful democratic running of the project empowered team members not only to suggest changes in design at each stage but also gave licence to concede, without retribution, when their earlier inputs were no longer relevant or workable. This resulted in mutual acceptance and learning from across the different disciplinary perspectives, leading to effective teamwork and sustainability of the project.

While there was democratic participation within the core team, we note that this was not always extended to visitors of the exhibit even though we considered them to be stakeholders in the process. After engaging with the exhibit, visitors were asked for their opinions in the pilot phase and debriefed on the experimental research, but prior to and during engagement with the exhibit, the purpose of the experimental conditions was withheld from visitors for risk of priming or biasing their creativity behaviour when interacting with the task. Thus, we note that our implementation of PAR had limits as the psychological nature of the experiment required that research was performed ‘on’, not ‘with’ visitors up until their interaction with the task was complete.

### Reflective practice, adapting and comprise

Reflection, flexibility, adaptability and accepting change are key components of any PAR project. The nature of PAR means that things can change quite quickly and often. There is therefore a need to be able to react quickly to changes to the project, but also to find the space to accommodate them whilst constantly referring back to and reflecting on the aims and objectives of the project. Reflective practice afforded us substantial insight into both academic and practitioner knowledge, norms and values regarding exhibit and experimental design. For example, from initially divergent understandings over the implementation and purpose of ethical consent, a user-friendly system suitable for rigorous data collection evolved.

Some of the academics were new to the lack of strictly defined outcomes (an aspect of the PAR approach) which served to release them from the pressure of requiring and implementing a known method for achieving a prescribed goal; more consistent with explorative rather than confirmatory scientific studies. Thus, as long as a team ensures that the original aims are not forgotten, outcomes may be far more innovative and appropriate than had they remained fixed from inception. For example, the comparison of a physical and digital version of the same task was not on the agenda at the beginning of the exercise but through the wide and far-reaching discussions involved in PAR it became a valuable, additional aim.

## Conclusion

Our PAR process appeared to effectively disrupt disciplinary boundaries, resulting in an innovative new blend of research practice and knowledge generation. Core team members gained confidence in undertaking research on the Science Centre floor and of the needs and agendas of those from different disciplinary backgrounds (both within and outside of academia/Science Centres). Moreover, the expertise of designing exhibits to digitally gather ethical consent from users and automatically digitally collect data, once consent is granted, has transformed the research capacity of both academics and Science Centre practitioners. Much as Whitman et al. highlighted the utility of PAR for physical geographers (Whitman, Pain, and Milledge 2015), we hope that this research (see creativescienceatlife.com for more information and developments) will highlight the utility of PAR and co-produced research between diverse academics and Science Centres. We have challenged traditional notions regarding how research is done and impact is achieved: our multidisciplinary team used PAR to enable co-produced research that iteratively developed knowledge and achieved impacts during the process, rather than impact being a separate stage of the project. The interactive research pod is a successful exhibit in terms of engaging visitors, and, providing a large sample size for research tasks contained within it. Our work aligns with a number of Pain and Raynor’s recent recommendations for consideration of co-production, and participatory, research as an alternative approach to impact. For our project societal impact was ‘at the core of why and how co-produced research takes place’ (Pain and Raynor 2016, 6). Moreover, the egalitarian, iterative and relatively open-ended, sometimes serendipitous, process of PAR that we found so beneficial, in generating novel impact, corresponds to the claim that, ‘The purpose of the research is what brings people together, it drives them and drives the twists and turns of the process’ (Pain and Raynor 2016, 6). Co-produced research (using PAR) challenges the current REF/RCUK model of impact, but is likely to be of interest to the innovative, and growing, engaged practice of Citizen Science and Citizen Observatories (Roy et al. 2012; Edwards, McDonnell, and Simpson 2016).

## Funding

This work was supported by an Economic and Social Research Council (ESRC) IAA award to RLK [grant number ES/M500586/1]; The Wellcome Trust for CfL’s Brain Zone [grant number WT103490AIA]; and a Slovenian Ad Futura studentship to Zarja Mursik (ZM).

## Disclosure statement

No potential conflict of interest was reported by the authors.

## References

[CIT0001] Argyris C. 2003 “Actionable Knowledge.” In *The Oxford Handbook of Organization Theory*, edited by Tsoukas H. and Knudsen C., 423–452. Oxford: OUP.

[CIT0002] Barak M., and Shachar A. 2008 “Projects in Technology Education and Fostering Learning: The Potential and Its Realization.” *Journal of Science Education and Technology* 17: 285–296. 10.1007/s10956-008-9098-2

[CIT0003] Barton A.C., Tan E., and Rivet A. 2008 “Creating Hybrid Spaces for Engaging School Science among Urban Middle School Girls.” *American Educational Research Journal* 45 (1): 68–103.

[CIT0004] Bonawitz E. 2011 “The Double-edged Sword of Pedagogy: Instruction Limits Spontaneous Exploration and Discovery.” *Cognition* 120 (3): 322–330. 10.1016/j.cognition.2010.10.001 21216395 PMC3369499

[CIT0005] Brown T. 2009 *Change by Design: How Design Thinking Transforms Organisations and Inspires Innovation*. New York: HarperBusiness.

[CIT0006] Cahill C. 2007 “Doing Research with Young People: Participatory Research and the Rituals of Collective Work.” *Children’s Geographies* 5 (3): 297–312. 10.1080/14733280701445895

[CIT0007] Carr K., Kendal R.L., and Flynn E.G. 2015 “Imitate or Innovate? Children’s Innovation is Influenced by the Efficacy of Observed Behaviour.” *Cognition* 142: 322–332. 10.1016/j.cognition.2015.05.005 26072278

[CIT0008] Chambers R. 2008 “PRA, PLA and Pluralism: Practice and Theory.” In *The Sage Handbook of Action Research*: *Participative Inquiry and Practice*, edited by Reason P. and Bradbury H., 297–318. London: Sage.

[CIT0009] Chevalier J.M., and Buckles D.J. 2008 *A Guide to Collaborative Inquiry and Social Engagement.*, *SAS2*. New Delhi: Sage.

[CIT0010] Chevalier J.M., and Buckles D.J. 2013 *Participatory Action Research: Theory and Methods for Engaged Inquiry*. Abingdon: Routledge.

[CIT0011] Coghlan D., and Brannick T. 2010 *Doing Action Research in Your Own Organisation*. 3rd ed. London: Sage.

[CIT0012] Cross N. 2011 *Design Thinking: Understanding How Designers Think and Work*. Oxford: Berg.

[CIT0013] Day R.L., MacDonald T., Brown C., Laland K.N., and Reader S.M. 2001 “Interactions between Shoal Size and Conformity in Guppy Social Foraging.” *Animal Behaviour* 62 (5): 917–925. 10.1006/anbe.2001.1820

[CIT0014] Defeyter M.A. 2014 “Children’s Understanding of Ownership Affects How Children Use Familiar Objects in Problem-solving Tasks.” Paper presented at the Understanding the Origins and Development of Human Creativity, St Andrews University, Fife, UK.

[CIT0015] DeWitt J., Archer L., and Osborne J. 2013 “Nerdy, Brainy and Normal: Children’s and Parents’ Constructions of Those Who Are Highly Engaged with Science.” *Research in Science Education* 43: 1455–1476. 10.1007/s11165-012-9315-0

[CIT0016] Dierking L.D., and Falk J.H. 2010 “The 95 Percent Solution: School is Not Where Most Americans Learn Most of Their Science.” *The American Scientist* 98 (6): 486–494.

[CIT0017] Edwards R., McDonnell D., and Simpson I. 2016 “Exploring the Relationship Between Educational Background and Learning Outcomes in Citizen Science”. Paper presented at the Citizen Science – Innovation in Open Science, Society and Policy, Berlin.

[CIT0018] Falk J.H., Dierking L.D., Swanger L., Staus N., Back M., Barriault C., Catalao C., et al 2016 “Correlating Science Center Use with Adult Science Literacy: An International, Cross-Institutional Study.” *Science Education* 100 (5): 849–876.

[CIT0019] Falk J.H., Storksdieck M., and Dierking L.D. 2007 “Investigating Public Science Interest and Understanding: Evidence for the Importance of Free-Choice Learning.” *Public Understanding of Science* 16 (4): 455–469. 10.1177/0963662506064240

[CIT0020] Frisby W., Reid C.J., Millar S., and Hoeber L. 2005 “Putting ‘Participatory’ into Participatory Forms of Action Research.” *Journal of Sport Management* 19 (4): 367–386.

[CIT0021] Gopnik A. 2012 “Scientific Thinking in Young Children: Theoretical Advances, Empirical Research, and Policy Implications.” *Science* 337 (6102): 1623–1627. 10.1126/science.1223416 23019643

[CIT0022] Halpern D.F., Eliot L., Bigler R.S., Fabes R.A., Hanish L.D., Hyde J., Liben L.S., and Martin C.L. 2011 “The Pseudoscience of Single-sex Schooling.” *Science* 333 (6050): 1706–1707. 10.1126/science.1205031 21940879

[CIT0023] HEFCE, SFC, HEFCW, and DELNI 2012 “Assessment Framework and Guidance on Submissions (02.2011 Updated to Include Addendum Published in January 2012)”. In *REF2014*. London.

[CIT0024] Hein G.E. 1999 “The Constructivist Museum.” In *The Educational Role of the Museum*, edited by Hooper-Greenhill E., 73–79. London: Routledge.

[CIT0025] Inhelder B., and Piaget J. 1958 *The Growth of Logical Thinking: From Childhood to Adolescence*. London: Routledge & Kegan Paul 10.1037/10034-000

[CIT0026] Kelley T. 2001 *The Art of Innovation: Lessons in Creativity from IDEO*. New York: Crown Business.

[CIT0027] Kendal R.L., Coolen I., and Laland K.N. 2004 “The Role of Conformity in Foraging When Personal and Social Information Conflict.” *Behavioural Ecology* 15 (2): 269–277. 10.1093/beheco/arh008

[CIT0028] Kidd J. 2014 *Museums in the New Mediascape: Transmedia, Participation, Ethics*. Ashgate: Surrey.

[CIT0029] King’s College London and Digital Science for HEFCE 2015 *The Nature, Scale and Beneficiaries of Research Impact: An Initial Analysis of Research Excellence Framework (REF) 2014 Impact Case Studies*. Bristol: HEFCE.

[CIT0030] Kirschner P.A., Sweller J., and Clark R.E. 2006 “Why Minimal Guidance during Instruction Does Not Work: An Analysis of the Failure of Constructivist, Discovery, Problem-based, Experiential, and Inquiry-based Teaching.” *Educational Psychologist* 41 (2): 75–86. 10.1207/s15326985ep4102_1

[CIT0031] Klahr D., and Nigam M. 2004 “The Equivalence of Learning Paths in Early Science Instruction: Effect of Direct Instruction and Discovery Learning.” *Psychological Science* 15 (10): 661–667. 10.1111/j.0956-7976.2004.00737.x 15447636

[CIT0032] Lane S.N., Odoni N., Landström C., Whatmore S.J., Ward N., and Bradley S. 2011 “Doing Flood Risk Science Differently: An Experiment in Radical Scientific Method.” *Transactions of the Institute of British Geographers* 36 (1): 15–36. 10.1111/tran.2010.36.issue-1

[CIT0033] Lennie J., and Tacchi J. 2013 *Evaluating Communication for Development: A framework for social change*. London: Routledge.

[CIT0034] Lewin K. 1946 “Action Research and Minority Problems” In *Resolving Social Conflicts: Selected Papers on Group Dynamics*, edited by Lewin G., 201–216. New York: Harper & Row.

[CIT0035] Lyons T. 2006 “The Puzzle of Falling Enrolments in Physics and Chemistry Courses: Putting Some Pieces Together.” *Research in Science Education* 36: 285–311. 10.1007/s11165-005-9008-z

[CIT0036] Macdonald A. 2014 “‘Not for People like Me?’ Under-represented Groups in Science, Technology and Engineering: A Summary of the Evidence: The Facts, the Fiction and What We Should Do Next.” In *Women in Science and Engineering Campaign*. Leeds: Leeds College.

[CIT0037] Martin L.M.W. 2004 “An Emerging Research Framework for Studying Informal Learning and Schools.” *Science Education* 88 (S1): S71–S82. 10.1002/(ISSN)1098-237X

[CIT0038] McWilliam E., Poronnik P., and Taylor P. G. 2008 “Re-designing Science Pedagogy: Reversing the Flight from Science.” *Journal of Science Education and Technology* 17: 226–235. 10.1007/s10956-008-9092-8

[CIT0039] NELEP 2014 *More and Better Jobs: The North East Strategic Economic Plan*. Newcastle-upon-Tynef: North East Local Economic Partnership.

[CIT0040] Newton L.D., and Newton D.P. 2010 “What Teachers See as Creative Incidents in Elementary Science Lessons.” *International Journal of Science Education* 32 (15): 1989–2005. 10.1080/09500690903233249

[CIT0041] Pain R., and Francis P. 2003 “Reflections on Participatory Research.” *Area* 35 (1): 46–54. 10.1111/area.2003.35.issue-1

[CIT0042] Pain R., and Raynor R. 2016 “Mapping Alternative Impact: Alternative Approaches to Impact from Co-Produced Research.” In, edited by Centre for Social Justice and Community Action. Durham University.

[CIT0043] Pain R., Kesby M., and Askins K. 2011 “Geographies of Impact: Power, Participation and Potential.” *Area* 43: 183–188. 10.1111/area.2011.43.issue-2

[CIT0044] Parry R. 2010 *Museums in a Digital Age*. Oxford: Routledge.

[CIT0045] Pedler M. 2005 *Action Learning in Practice*. Farnham: Gower Publishing.

[CIT0046] Rahm J. 2014 “Reframing Research on Informal Teaching and Learning in Science: Comments and Commentary at the Heart of a New Vision for the Field.” *Journal of Research in Science Teaching* 51 (3): 395–406. 10.1002/tea.v51.3

[CIT0047] Reason P., and Bradbury H. 2008 *Handbook of Action Research*. 2nd ed. London: Sage 10.4135/9781848607934

[CIT0048] Roy H.E., Pocock M.J.O., Preston C.D., Roy D., Savage J., Tweddle J.C., and Robinson L.D. 2012 “Understanding Citizen Science and Environmental Monitoring.” In NERC Centre for Ecology & Hydrology and Natural History Museum.

[CIT0049] Sacco K., Falk J.H., and Bell J. 2014 “Informal Science Education: Lifelong, Life-wide, Life-deep.” *PLOS Biology* 12 (11): 1–3. 10.1371/journal.pbio.1001986 PMC4219654 25369429

[CIT0050] Sandifer C. 2003 “Technological Novelty and Open-endedness: Two Characteristics of Interactive Exhibits That Contribute to the Holding of Visitor Attention in a Science Museum.” *Journal of Research in Science Teaching* 40 (2): 121–137. 10.1002/(ISSN)1098-2736

[CIT0051] Schmidt A. 2011 “Creativity in Science: Tensions between Perception and Practice.” *Creative Education* 2: 435–445. 10.4236/ce.2011.25063

[CIT0052] Schon D. 1983 *The Reflective Practitioner: How Professionals Think in Action*. New York: Basic Books.

[CIT0053] Shura R., Siders R.A., and Dannefer D. 2011 “Culture Change in Long-term Care: Participatory Action Research and the Role of the Resident.” *The Gerontologist* 51 (2): 212–225. 10.1093/geront/gnq099 21163911 PMC3140257

[CIT0054] Straw S., MacLeod S. 2013 “Improving Young People’s Engagement with Science, Technology, Engineering and Mathematics (STEM).” In *NFER Thinks: What the Evidence Tells Us*, edited by National Foundation for Educational Research in England and Wales, 1–5. Slough: NFER.

[CIT0055] Tallon L., and Walker K. 2008 *Digital Technologies and the Museum Experience: Handheld Guides and Other Media*. Lanham, MD: Altamira Press.

[CIT0056] UK Commission for Employment and Skills 2014 *UK Labour Market Projections: 2014 to 2024* London: Gov.uk.

[CIT0057] Vygotsky L.S. 1978 *Mind in Society: The Development of Higher Psychological Processes*. Harvard: Harvard University Press.

[CIT0058] Warrior J. 2002 “Singularly Successful: Report on Teaching Science and Technology in Single Sex Classes in Co-educational School.” Report for Women in Science and Engineering Campaign Leeds: Leeds College.

[CIT0059] Whitman G., Pain R., and Milledge D. 2015 “Going with the Flow? Using Participatory Action Research in Physical Geography.” *Progress in Physical Geography* 39 (5): 622–639. 10.1177/0309133315589707

[CIT0060] Zhang S., Schmader T., and Forbes C.E. 2009 “The Effects of Gender Stereotypes on Women’s Career Choice: Opening the Glass Door” In *The Glass Ceiling in the 21st Century: Understanding Barriers to Gender Equality*, edited by Barreto M., Ryan M.K., and Schmitt M. T., 21–47. Washington, DC: American Psychological Association.

